# Safety culture in the polish mining and extractive sector: trend analysis for 2019–2025

**DOI:** 10.3389/fpubh.2026.1778898

**Published:** 2026-06-10

**Authors:** Mariusz Kapusta, Patrycja Bąk, Marta Sukiennik

**Affiliations:** Faculty of Civil Engineering and Resource Management, AGH University of Krakow, Krakow, Poland

**Keywords:** hard coal mine, hazard assessment, mining enterprise, occupational health and safety (OHS), organizational culture, safety culture grid

## Abstract

This study analyzes occupational safety culture in the Polish hard coal mining sector, based on surveys conducted in 2019 and repeated in 2025 among managerial staff in three major mining companies. The research utilized expert diagnostics and audit questionnaires, covering seven key areas of safety culture, including leadership, risk analysis, safety briefings, and regulatory compliance. Results from 2019 revealed deficits in management participation and adherence to organizational and legal regulations, with a workforce skewed toward mid- and long-term tenure, indicating a potential generational gap. By 2025, improvements were observed across most areas, particularly in leadership, vision, and safety briefings, reflecting greater engagement and a proactive approach to occupational health and safety. Minor decreases in some areas were linked to workforce reductions and restructuring. Benchmarking against three safety culture segments indicated a shift from the Responsibility-Based Organization level in 2019 to the Responsibility-Oriented Safety Culture level in 2025, demonstrating the integration of safety as a core organizational value. The study highlights the importance of managerial leadership, employee engagement, and systemic measures in sustaining and further developing workplace safety culture in mining enterprises. The survey conducted in 2025 aimed to assess changes in employees’ attitudes, behaviors, and safety awareness related to occupational health and safety.

## Introduction

The issue of organizational culture has been the subject of interest for many researchers worldwide and concerns a wide range of activities and various types of economic entities (1–4). Contemporary scholars from numerous countries are increasingly focusing on the study of safety culture in underground mines, as it constitutes a specific dimension of organizational culture related to occupational health and safety. In this context, safety culture should be understood as a subset of organizational culture that reflects how safety is perceived, valued, and managed within an organization A mature safety culture is characterized not only by compliance with safety procedures but also by deeply internalized safety values, proactive attitudes, and continuous improvement in risk management practices, which together contribute to improved outcomes in reducing the number of accidents (5). An individualized approach to employee protection, while maintaining operational efficiency, involves, among other aspects, the enhancement of occupational safety in coal mines (6). Therefore, it is necessary to develop a dedicated cultural model tailored to the specific operating conditions of each enterprise (7).

Organizational culture is one of the key factors influencing the functioning and development of an enterprise. Numerous definitions presented in the literature contain concepts and descriptions that either correspond to or explicitly define organizational culture. Among them are references to symbols, communication patterns, rituals, myths, and the organizational climate (8–10).

Organizational culture is such a complex concept that it can be defined in numerous ways. In the literature, several additional approaches can also be found, including the following:

- “Organizational culture can have a significant impact on digital transformation. The authors examine the impact of organizational culture on digital transformation by identifying the cultural values that influence digital transformation and the ways in which they support a successful digital transformation.” (11)
- “Organizational culture is the way in which organizations operate; organizational culture is the sum of the values and rituals that bind the organization’s members together; organizational culture is the culture of the workplace.” (12)
- “Organizational culture encompasses deeper values and serves as the foundation for developing shared organizational norms.” (13)
- “Other authors view organizational culture as a set of values and norms, attitudes and codes of conduct that an organization’s employees adhere to when carrying out their business activities.” (14–17).
- “Organizational culture encompasses socio-cultural activities, recurring patterns of perception, methods of working and assessing organizational culture, as well as sets of myths, symbols and common behaviors.” (18–20)
- “Organizational culture consists of unwritten rules, often perceived subconsciously, which bridge the gap between what is unspoken and what actually happens. It concerns shared views, ideologies, values, beliefs, expectations and norms.” (9)
- “Culture is a set of norms and values followed by employees. It encompasses a hierarchy of values, remuneration, career development, loyalty and authority, participation, mutual communication, and innovativeness.” (21)
- “Organizational culture constitutes a system of values, traditions, aspirations, beliefs and attitudes that form the essence of everything that is done and thought within an organization. It is sustained by a system of rituals, ceremonies, communication patterns and informal structures.” (22)
- “Culture may be defined as the beliefs and convictions disseminated within an organization concerning how business should be conducted, how employees should behave, and how they should be treated.” (23)

The most frequently cited definition and interpretation of organizational culture is Schein’s model, widely recognized as a framework that systematizes the concept of culture. Schein (24) employed the metaphor of an iceberg to illustrate the elements he distinguished. He identified three levels of organizational culture: underlying assumptions, beliefs and values, and artifacts.

This model is referred to as the iceberg model because, although artifacts are the most visible components, the true drivers of behavior and attitudes within a given community are the underlying assumptions, which cannot be easily observed or understood without knowledge of the deeper cultural code.

The complexity of organizational culture, along with the multiplicity of its determinants and classifications, makes both its examination and measurement ambiguous. The literature proposes numerous approaches to address these challenges, and similarly to the difficulties in defining organizational culture itself, a wide variety of methodological solutions exist.

In summary, organizational culture is not homogeneous, nor are the research approaches and methods used to measure it. Therefore, to ensure accurate assessment, it appears necessary to apply those methods and tools that are best suited to the specific characteristics of a given organization. Equally important are the practical feasibility of research implementation and the availability of measurement tools. A commonly applied solution is the integration of multiple instruments, combining both quantitative and qualitative methods to assess organizational culture.

Organizational culture provides the broader framework within which specific cultural dimensions emerge, including safety culture.

Among the various dimensions of organizational culture discussed in the literature, particular attention is given to safety culture, which is widely recognized as a specific manifestation of organizational culture related to occupational health and safety. While organizational culture encompasses a wide range of values and practices related to overall organizational functioning, safety culture focuses specifically on issues related to risk, safety behaviors, and the prioritization of health and safety in everyday operations. Safety culture reflects the shared values, beliefs, attitudes, and behavioral patterns that determine how safety issues are perceived, prioritized, and managed within an organization. In high-risk industries, such as mining and the extractive sector, this dimension of organizational culture becomes particularly significant, as employees’ attitudes toward safety, compliance with procedures, and communication regarding hazards may directly influence the likelihood of accidents and the overall level of occupational risk. For this reason, many authors addressing occupational safety issues first refer to the broader concept of organizational culture and subsequently focus on the more specific concept of safety culture.

The importance of this issue is particularly evident in industries characterized by a high level of occupational risk. One such sector is the extractive industry, where the specificity of working conditions and the work environment generates numerous hazards.

The Polish extractive industry—particularly work conducted in underground mines—is determined by numerous factors. Geological and mining conditions, together with technical and organizational factors, constitute two primary groups that generate risk. The progressive deepening of hard coal extraction poses new challenges for the mining industry, primarily through the emergence of additional occupational hazards in the underground work environment (25). Historically, the most severe disasters in global hard coal mining have been associated with coal dust and methane explosions (26, 27). However, the literature also indicates broader classifications of causes and hazards related to accidents in coal mines (28).

This study contributes to the literature in three key ways. First, it provides one of the few longitudinal analyses of safety culture in the European mining sector, capturing changes over a six-year period under conditions of significant socio-economic transformation. Second, it integrates the safety culture grid with expert diagnostics, offering a hybrid methodological approach that enables both structural and behavioral assessment of safety culture. Third, the study contributes to organizational culture theory by empirically demonstrating the role of managerial leadership in the transition from responsibility-based to responsibility-oriented safety culture. From a practical perspective, the findings provide actionable insights for mining enterprises undergoing restructuring and energy transition.

## Methods

### Safety culture measurement

In the international literature, numerous publications address occupational safety culture. Most authors adapt its characteristics to the specific legal, economic, organizational, and mining-geological conditions existing in individual countries. A common feature, however, is its strong association with management processes and the assessment of occupational risk (29). Research in this field typically involves the development of questionnaires or surveys administered to a representative sample of employees working in mining enterprises (30). The information obtained in this way often enables the management of underground mines to develop sets of rules that ensure effective compliance with safety regulations (31).

Safety culture may also be considered an indicator of the degree to which safety standards and regulations are implemented and understood within an organization (32). In this context, appropriate methods for its measurement are required (33). One commonly applied method of measuring workplace safety culture is the expert interview, which allows the identification of specific behavioral conditions of employees within an organization (34). The results of such studies make it possible to analyze employee errors in enterprises and to determine future research directions (35).

**Figure 1:**
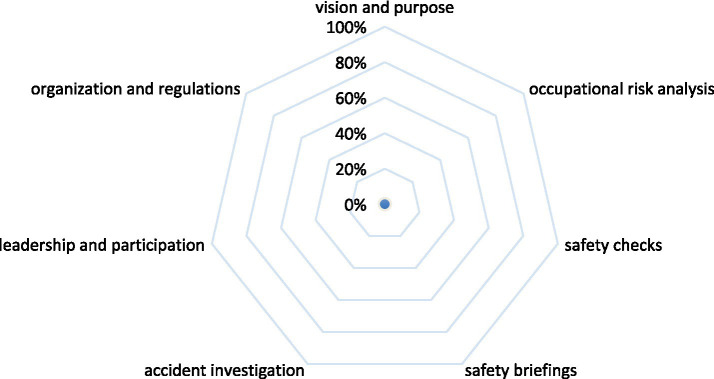
Safety culture grid—source: authors’ own study based on Sukiennik et al. (36).

Another method used to assess occupational safety culture is the so-called safety culture grid (Figure 1). Its purpose is to identify both the strengths and the weaker aspects of safety culture within the analyzed enterprise. It also aims to indicate what measures should be implemented by the company in order to strengthen the level of safety culture within its organizational structures.

The safety culture grid has the form of a heptagon, with its vertices representing factors that determine the level of safety culture within an enterprise. These include:

- Vision and purpose;
- Occupational risk analysis;
- Safety checks;
- Safety briefings;
- Accident investigation;
- Leadership and participation;
- Organization and regulations.

The selected dimensions correspond to the key components of the safety culture grid and reflect critical areas of safety management, including leadership, risk assessment, communication, and organizational practices.

Values on the grid are expressed as percentages. Each factor is assigned a specific value resulting from the characteristics of that element in the assessed enterprise. This value is then marked as a point on the grid. Once point values have been determined for all factors, an area is generated that reflects the level of safety culture within the organization.

The curve formed by connecting individual points provides information on the percentage contribution of each factor to shaping the level of safety culture in the enterprise.

Another tool that can assist in determining the level of occupational health and safety (OHS) culture is benchmarking. In a comparative analysis (benchmarking), safety culture can be divided into three segments (36):

- Convenience-Driven organization—where safety remains primarily the responsibility of individual specialists, and is not yet fully embedded in organizational processes;
- Responsibility-Based organization—in which adequate organizational structures exist, but safety is still regarded as a task for management. Consequently, not all safety-related activities are always carried out in accordance with established procedures;
- Responsibility-Oriented Safety Culture—a situation in which occupational health and safety are fully integrated into management responsibilities, while employee behavior becomes increasingly self-accountable. In this model, accidents are perceived as weaknesses in the safety system.

**Figure 2:**
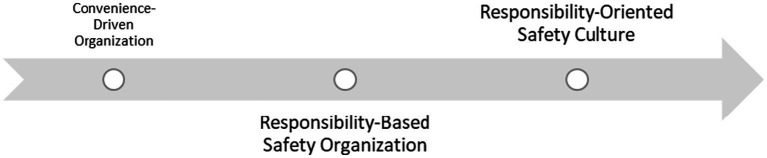
Safety culture segments—source: authors’ own study based on Kirschstein and Werner-Keppner (43).

This categorization can be illustrated incrementally to reflect the levels of enterprise adaptation to safety culture (Figure 2).

The lowest level in the discussed classification is the so-called Convenience-Driven Organization, characterized by a low degree of implementation of OHS principles and limited employee engagement with safety culture issues. At this stage, OHS-related activities are usually fragmentary and incidental, often limited to fulfilling formal legal requirements. There is also a lack of a systematic approach to safety management, resulting in insufficient effectiveness of the implemented measures.

The next level of development is referred to as the Responsibility-Based Organization. In this model, the organization develops and implements structured occupational safety rules, and in many cases establishes integrated safety management systems. Employees are aware of the consequences of non-compliance with existing procedures and generally adhere to them. However, this compliance often stems primarily from imposed organizational rules rather than from individual, internalized responsibility. Nevertheless, this level represents an important transitional stage between mere formal compliance and the establishment of a mature safety culture.

The highest level corresponds to Responsibility-Oriented Safety Culture. Achieving this stage indicates a high degree of organizational maturity, where both management structures and employees are fully prepared to consistently comply with OHS principles. In organizations operating at this level, employees demonstrate conscious, autonomous, and proactive engagement in maintaining and improving safety standards. Importantly, OHS activities are systematic, continuous, and improvement-oriented. Initiatives are planned, implemented, and monitored to promote the development of safety culture, driven both by business considerations—such as reducing the number of accidents, stabilizing production processes, and cutting costs—and by humanitarian con The analysis of the presented classification clearly indicates that the strategic goal of every organization should be the achievement of a Responsibility-Oriented Safety Culture. This is particularly important in sectors of the economy where occupational hazards are pronounced and characterized by a high level of risk. Such sectors include, in particular, the extractive industry, especially hard coal mining, where workplace safety is determined not only by organizational and technical factors but also by geological and mining conditions.

In this context, it is crucial for mines to self-assess their position within the presented classification of safety culture levels. Conducting a thorough self-evaluation enables the identification of areas requiring improvement, the targeting of preventive measures, and more effective implementation of strategies to enhance workplace safety. Consequently, this can contribute to a sustained improvement in safety indicators and the systematic advancement of OHS culture within mining organizations.

To date, systematic and repeatable studies aimed at assessing the level of workplace safety culture have not been conducted in Polish mining. Yet, proper identification and analysis of key elements of safety culture can significantly contribute to reducing risky behaviors among employees (37). In recent years, mining enterprises have primarily focused on implementing occupational safety management systems, integrating them with existing quality and environmental management systems. It has been noted that an individualized approach to the role of employees in shaping safety culture helps reduce the likelihood of recurrent accidents (38). Moreover, a mature safety culture is recognized as a key factor in reducing occupational morbidity and improving employee health (5). Consequently, there remains a need for modern and innovative research methods that can identify factors affecting workplace safety and facilitate more effective accident prevention in the mining sector (39), as well as address issues related the protection of workers’ health and safety (40).

The definition of safety culture in mining enterprises largely results from the system of values, attitudes, and behaviors of management staff, particularly managers and direct supervisors. The role of this group is especially significant, as they have the greatest influence on the development of appropriate procedures and management tools, including those related to the creation of safe and hygienic working conditions (41). Previous research in this area has primarily focused on three aspects: tools supporting the development of safety culture, the role of management and supervisors in shaping it, and the attitudes and behaviors of employees (42).

In practice, the mining sector predominantly adopts a pragmatic approach, in which safety culture is primarily understood as the organization’s response to occupational risk. This is manifested in the ability to learn from incidents and accidents, anticipate hazards, and implement preventive measures (1). An important tool for shaping attitudes and reinforcing safety culture is employee education. This is achieved, among others, through postgraduate studies and specialized vocational courses, which not only enhance knowledge and competencies but also provide the qualifications necessary to work in OHS services. In many mining enterprises, tailored training programs and postgraduate studies in occupational health and safety, commissioned directly by employers, constitute a key element of safety management strategies (2).

Despite the growing body of literature on safety culture, there is still a lack of longitudinal studies examining changes in safety culture over time, particularly in the context of the Polish extractive industry. Existing research is often limited to cross-sectional analyses or focuses on selected aspects of safety culture, without capturing its dynamic nature. Therefore, this study aims to address this gap by providing a comparative analysis of safety culture based on empirical data collected in 2019 and 2025 in major underground mining enterprises.

In 2019, the authors conducted a study on the level of safety culture in three hard coal companies using an audit survey technique. The selection of companies was purposeful, encompassing enterprises employing approximately 90% of the workforce in the entire Polish coal mining sector. The study adopts a diagnostic and exploratory approach, aimed at identifying strengths and weaknesses in safety culture rather than testing causal relationships, which is consistent with commonly applied methods in organizational culture research.

The survey research was preceded by a diagnostic inquiry, referred to as the expert method, conducted individually with OHS staff and employee safety representatives in the mining enterprises. Based on the consultations and the authors’ experience from previous studies, a set of 120 items was created, relating to experts’ declared beliefs and opinions regarding workplace safety in mines.

The questionnaire consisting of 120 items was developed based on a review of the literature on safety culture and occupational health and safety, as well as expert consultations conducted with OHS specialists and practitioners in the mining sector. The items were designed to reflect key dimensions of safety culture identified both in previous research and in industry practice.

In 2025, the authors repeated the study—the same survey was administered to employees of the same companies. The aim was to examine how results had changed over several years, how external determinants had shaped the current state of safety culture in mines, and, ultimately, whether changes in recent years had influenced employees’ awareness of occupational safety.

## Results

### Research in coal mines

The study was conducted in 2019 among employees holding managerial positions (mining supervision) who were randomly selected for the sample.

The study focused on managerial staff due to their key role in shaping safety culture, decision-making processes, and implementation of OHS policies. While this approach may limit generalizability to operational workers, it allows for capturing strategic and organizational dimensions of safety culture.

**Figure 3:**
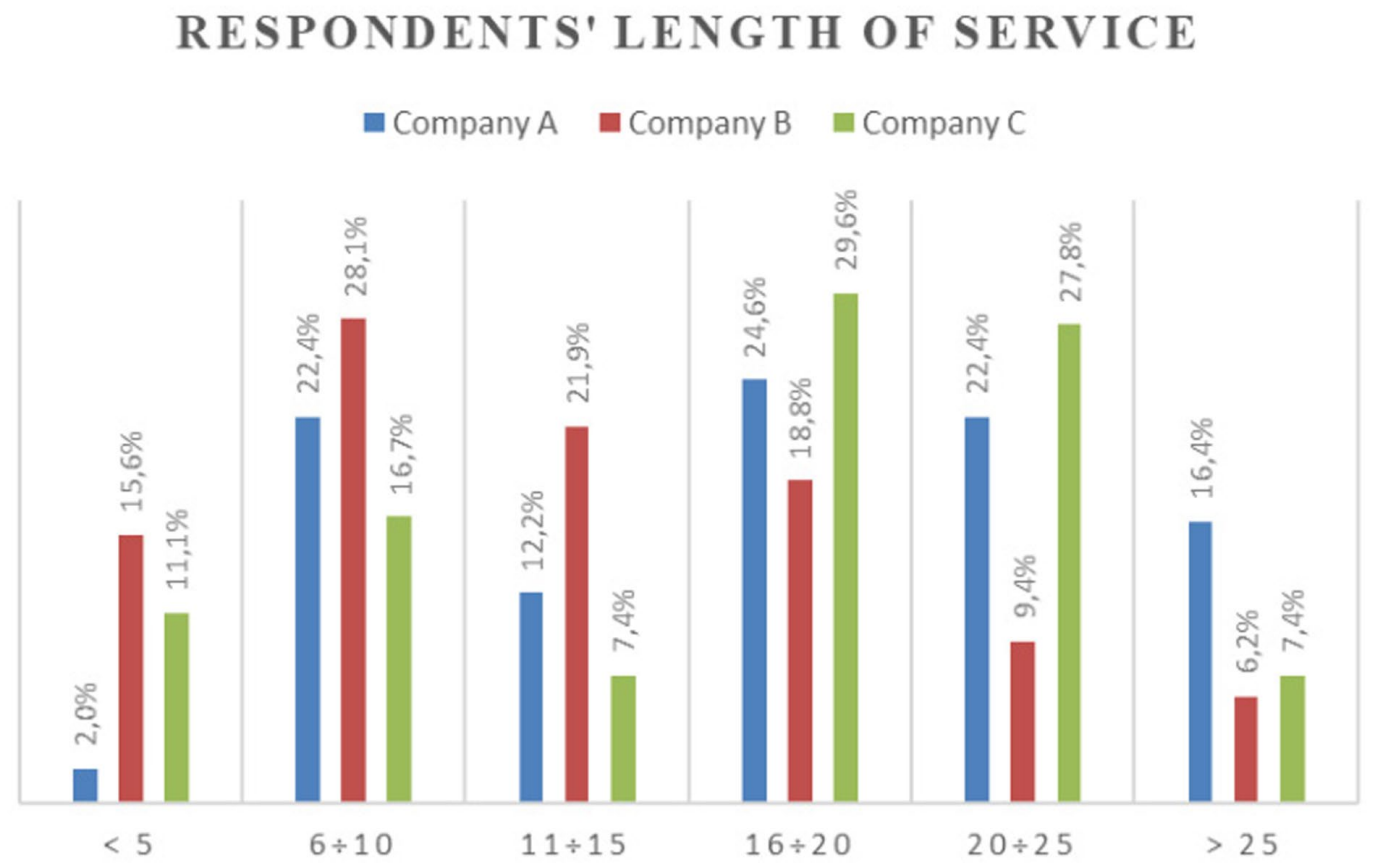
Respondents’ length of service in the studied mining companies in 2019. Source: authors’ own study.

The variation in workplace hazards across positions in the different company sites may undoubtedly influence perceptions of safety issues. A total of 135 employees completed the questionnaires, proportionally to the total number of staff employed in each coal company. Figure 3 shows the respondents’ professional experience.

Based on the obtained results, it is noteworthy that in 2019 the majority of employees had between 16 and 20 years of work experience, accounting for 25.2% of respondents. Of particular concern, however, is the finding that only 8.9% of respondents had up to 5 years of experience, which may lead to a “generational gap” in the future.

**Figure 4:**
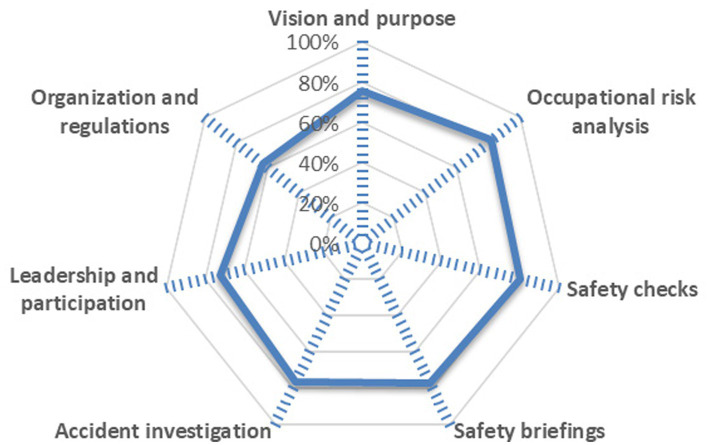
Safety culture grid for the studied mining sector in 2019. Source: authors’ own study.

Using the data obtained from the study, radar charts were created to illustrate the level of safety culture across the analyzed assessment areas. Figure 4 presents the Safety Culture Grid of the surveyed mining enterprises in 2019, providing a visual representation of the results obtained.

**Figure 5:**
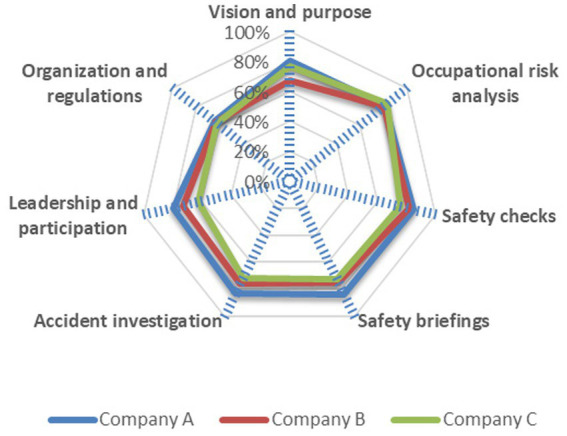
Safety culture grid in individual mining companies in 2019. Source: authors’ own study.

The next chart (Figure 5) illustrates the Safety Culture Grid with a breakdown by individual mining companies. According to the authors’ approach, the combined diagnostic and analytical method enables the identification of strong and weak areas of safety culture (3, 45). This allows for an attempt to interpret identified adverse phenomena and to draw conclusions aimed at detecting dysfunctional areas. The findings obtained from the study should serve as guidance for improving workplace safety.

To verify the method used for measuring occupational safety culture in hard coal mines in 2019, the authors repeated the study in 2025. It is noteworthy that during this period, socio-political changes occurred in Poland that significantly influenced the attitudes and behaviors of mining enterprise employees. The state of epidemic threat and the state of epidemic were in effect in Poland from March 14, 2020, to June 30, 2023. This social situation caused multi-week interruptions in production processes as well as employment restrictions. For hard coal mines, this meant halting mineral extraction and minimizing the number of employees in the mines to the essential personnel required to ensure the functioning of the enterprise (2).

A key factor was also the political aspect related to the Russian Federation’s aggression against Ukraine, which began on February 24, 2022. Over the following months, severe energy problems arose due to restrictions on raw material imports from Russia. During this time, there was a sharp increase in electricity and heat prices, which significantly affected the economic situation of mining enterprises.

Another challenge for Polish coal mines is achieving climate neutrality targets, which are closely linked to the energy transition pursued within the European Union. The reduction of greenhouse gas emissions, implemented through programs limiting energy production from fossil fuels, reduces the demand for hard coal. In 2024, the share of electricity production from renewable energy sources reached 29.4%, simultaneously causing a 56.2% decrease in coal-based production. It is worth noting, however, that according to the assumptions of the national energy policy, the planned share of coal in electricity generation was to reach 56% by 2030. A key element of Poland’s energy system transformation was to be nuclear energy. According to these assumptions, the first block of a nuclear power plant was planned to be commissioned in 2033, with several additional blocks to follow gradually until 2036 (44).

**Figure 6:**
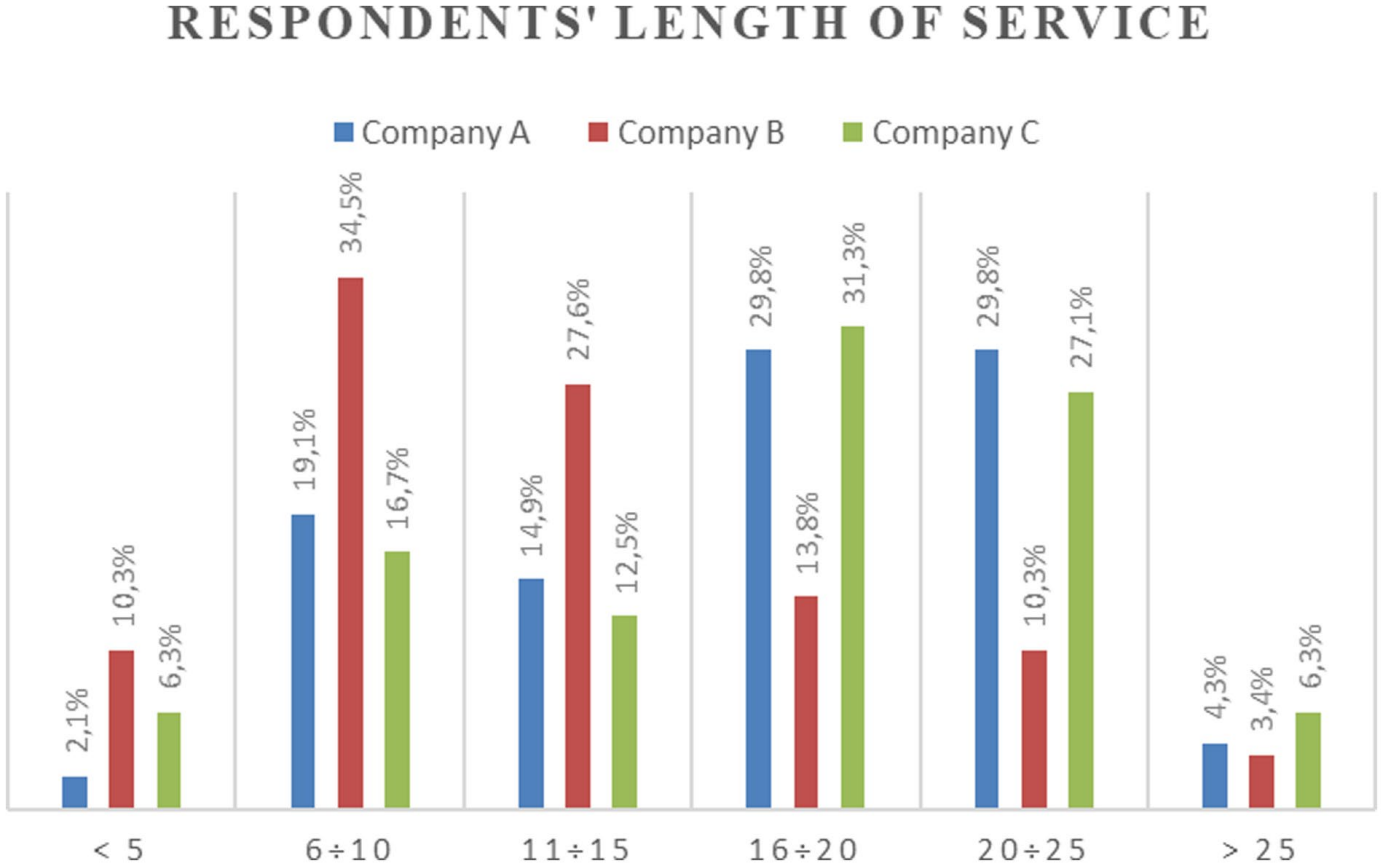
Respondents’ length of service in the studied mining companies in 2025. Source: authors’ own study.

The 2025 study was conducted based on the methodology proposed in 2019. In this case, responses were also provided by employees holding managerial positions. A total of 124 employees completed the questionnaires, in proportions similar to the total number of staff employed in each mining company. Figure 6 present the respondents’ work experience.

It can be observed that there was a noticeable decline in both the group of employees with up to 5 years of experience and the group with more than 25 years of experience. The process of energy transition, as well as the restructuring of the Polish hard coal mining sector, resulted in a suspension of employment for employees with lower work experience during the analyzed period. Furthermore, employees with more than 25 years of experience, according to regulations, acquire retirement rights and, upon leaving their positions, contribute to an accelerated reduction in the workforce within the mines.

**Figure 7:**
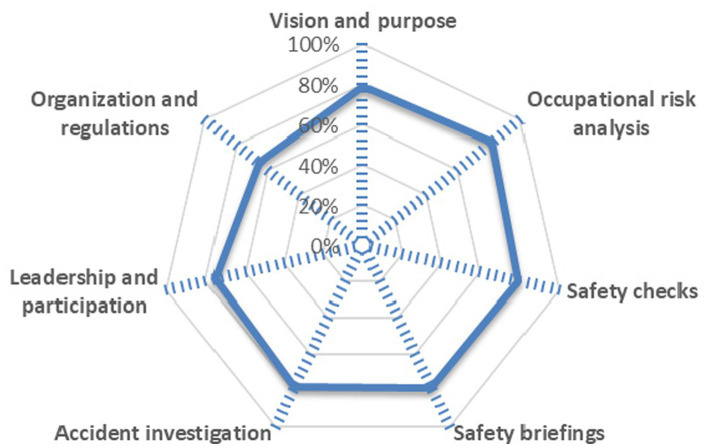
Safety culture grid for the studied mining sector in 2025. Source: authors’ own study.

**Figure 8:**
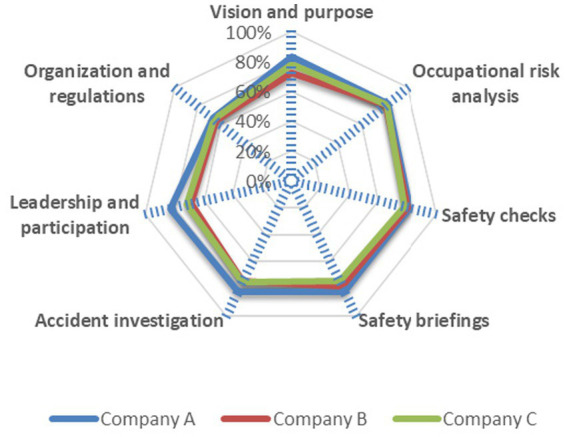
Safety culture grid in individual mining companies in 2025. Source: authors’ own study.

The repeated study in 2025 demonstrates observable changes. To clearly highlight these, Figures 7, 8 presents the results of the survey conducted in the various areas of the Safety Culture Grid for 2025, with a breakdown by individual companies.

The analysis of the study results indicates that the greatest deficits are observed in two areas:

- The approach to work organization and legal regulations,
- Management and participation in safety-related matters.

In the detailed surveys, a five-point Likert scale was used, allowing the assessment of safety culture at three levels. Graphical visualization using radar charts significantly facilitates the interpretation of the results.

## Discussion and conclusions

Mining remains an industry in which the elimination of certain risk factors is inherently limited. Therefore, continuous efforts are required to improve OHS conditions and to strengthen safety culture at both organizational and operational levels.

The comparative analysis of results from 2019 and 2025 indicates a generally positive trend in the development of safety culture in the studied mining enterprises. Improvements can be observed across most assessed dimensions. In particular, increases were noted in leadership and participation (from 72.4% in 2019 to 75.3% in 2025) and in organization and regulations (from 63.2 to 66.0%), which suggests a gradual strengthening of managerial engagement and a more structured approach to safety management. A moderate improvement was also observed in vision and purpose (from 75.3 to 78.6%), indicating a clearer strategic orientation toward safety-related objectives.

At the same time, some areas did not show equally strong progress. For example, safety checks slightly decreased (from 80.4 to 79.6%), which may reflect organizational changes and workforce restructuring during the analyzed period. These differences suggest that while strategic and leadership dimensions of safety culture are improving, some operational practices may require further reinforcement.

**Figure 9:**
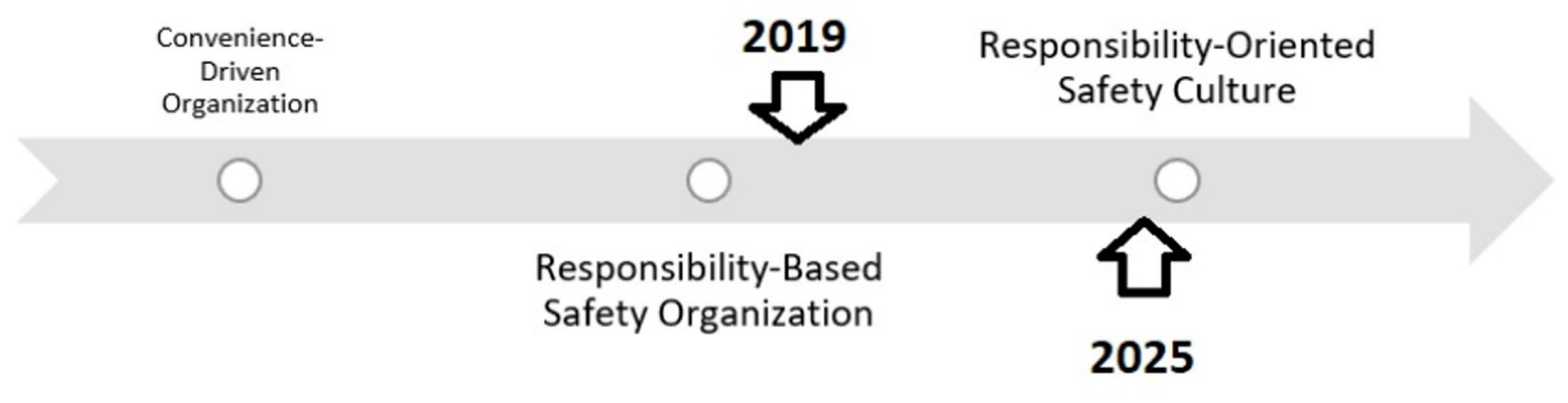
Safety culture segments in Polish mining sector in 2019 and 2025 culture segments—source: authors’ own study.

Referring to the safety culture classification, the results indicate a shift from the Responsibility-Based Organization level in 2019 toward the Responsibility-Oriented Safety Culture level in 2025. This transition is supported by the observed increases in key dimensions related to leadership, employee engagement, and organizational practices, suggesting a growing internalization of safety as a core organizational value. This shift is illustrated in Figure 9.

An important observation is the consistency of trends across the analyzed companies. Despite differences in individual results, all entities demonstrate a similar direction of change, which may indicate a broader, sector-wide transformation influenced by external factors such as organizational restructuring, energy transition, and socio-economic conditions.

Despite these positive developments, the results also indicate that certain areas—particularly organization and regulations (66.0% in 2025)—remain relatively weaker compared to other dimensions. This suggests that further efforts are required to strengthen formal structures, compliance mechanisms, and consistency in safety-related procedures.

The findings confirm that managerial staff play a critical role in shaping safety culture, particularly in high-risk industries such as mining. Their influence on decision-making processes, communication, and implementation of OHS policies directly affects the level of safety culture within the organization.

Despite the valuable insights provided by this study, certain limitations should be acknowledged. One limitation of the study is the use of descriptive analysis without applying inferential statistical tests. While the adopted approach is consistent with the diagnostic nature of the safety culture grid method, future studies could incorporate statistical techniques to test the significance of observed changes and further strengthen the robustness of conclusions. Additionally, the study focused exclusively on managerial staff, which may limit the generalizability of the findings. Future research should therefore include operational employees to provide a more comprehensive perspective on safety culture across organizational levels. Additionally, the questionnaire did not undergo formal statistical validation procedures (e.g., construct validity testing or reliability analysis), which constitutes a limitation of the study. However, the instrument was developed based on expert knowledge and prior research, ensuring its practical relevance and content adequacy.

In conclusion, the study demonstrates that safety culture in the Polish mining sector has evolved in a positive direction between 2019 and 2025. However, maintaining and further strengthening this trend requires continued organizational commitment, particularly in areas related to formal structures, regulatory compliance, and operational safety practices.

## Data Availability

The raw data supporting the conclusions of this article will be made available by the authors, without undue reservation.
